# A new fixation method for anterior cruciate ligament femoral avulsion fracture: a rare case report and literature review

**DOI:** 10.3389/fsurg.2025.1501740

**Published:** 2025-04-08

**Authors:** Jiquan Shen, Liang Hong, Changjian Zhou, Xinggao Wang, Zhijun Ye, Bo Wang

**Affiliations:** ^1^ Department of Orthopaedics, The Sixth Affiliated Hospital of Wenzhou Medical University, The First Affiliated Hospital of Lishui University, The People’s Hospital of Lishui, Lishui, Zhejiang, China; ^2^ Department of Orthopaedics, The People’s Hospital of Yunhe, Lishui, Zhejiang, China

**Keywords:** ACL injury, femoral avulsion fracture, arthroscopy, case report, knee injuries

## Abstract

**Purpose:**

To address the clinical challenges of femoral avulsion fractures of the anterior cruciate ligament (ACL), which are rare and lack a consensus on optimal treatment, through the presentation of a novel minimally invasive arthroscopic technique.

**Methods:**

An 18-year-old female with an ACL femoral avulsion fracture and a medial collateral ligament (MCL) rupture underwent arthroscopic anchor stitching using a composite absorbable bone anchor. This technique aimed to achieve anatomical reduction and support early functional recovery.

**Results:**

The surgical intervention achieved successful anatomical reduction. At the 6-month follow-up, the patient exhibited full knee mobility, joint stability, and resumed normal activities without discomfort. By the final 17-month follow-up, computed tomography (CT) confirmed complete fracture union, with preserved joint architecture and no degenerative changes. The knee remained stable and pain-free, demonstrating sustained efficacy of the technique.

**Conclusion:**

The arthroscopic anchor stitching technique is a viable, minimally invasive option for ACL femoral avulsion fractures, promoting rapid recovery and excellent long-term outcomes. This case highlights the importance of early recognition and anatomical fixation for such injuries.

## Introduction

Anterior cruciate ligament (ACL) injuries are among the most prevalent knee ligament injuries, occurring approximately once in every 3,500 people (1, 2). However, avulsion fractures at the ACL attachment are extremely rare. In children and adolescents, avulsion fractures are more common than pure ligament injuries due to their ligaments being stronger relative to their still-developing bone attachment points (3, 4). Most avulsion fractures affect the tibial side rather than the femoral side, although the exact reasons for this disparity remain unclear (5). To date, there have been 17 reported cases of ACL femoral avulsion fractures in pediatric patients and only 6 among adults (6–28). While there is no consensus on the optimal treatment, surgical intervention is generally recommended to promote fracture healing and restore ligament function, although alternative treatments may be considered for very young or elderly patients (11).

Here, we report the case of an 18-year-old female who sustained an ACL femoral avulsion fracture, along with a rupture of the femoral attachment of the medial collateral ligament (MCL). Her skeletal development was complete at the time of injury. We utilized an innovative arthroscopic anchor stitching technique to fix the ACL avulsion fracture and utilized absorbable sutures to repair the MCL. Additionally, we summarize previously reported cases of ACL femoral avulsion fractures (Tables 1, 2). The patient and her parents were fully informed and provided their verbal understanding and written consent prior to the submission of case information.

**Table 1:** Published cases of adult anterior cruciate ligament femoral avulsion fractures.

Research	Publication		Year	Age	Sex	Cause	Knee joint combined injury	Treatment methods	Outcome
Nagaraj (6)	The Southeast Asian Journal of Case Report and Review	2015		20	M	Motorcycle accident	LM tear	Arthroscopy with BPTB ACL reconstruction	3-month, negative Lachman test, 0–135° ROM.
Prasathaporn (7)	Arthroscopy Techniques	2015		25	M	Slipped	/	Arthroscopy, Fixed with a suture anchor	6-month, negative Lachman, anterior drawer, and pivot-shift tests.
Shah (8)	Radiology Case Reports	2015		47	F	Hit by a car	Open right tibial plateau and proximal fibula fractures	Arthroscopy, fixed with a 4-mm cancellous screw and washer	/
Zabiere (9)	Knee Surgery, Sports Traumatology, Arthroscopy	2016		50	M	Spinning sprain	/	Arthroscopy, fixed with two 2.4 mm cannulated screws.	14-month, negative Lachman test, stable and painless knee with full motion.
Tharakulphan (10)	BMJ Case Reports	2018		32	M	Non-contact pivoting injury	/	Arthroscopy, Pull-out sutures	5-month, negative Lachman test, returned to pre-injury activities.
Brandsma (11)	Journal of Surgical Case Reports	2020		60	F	Falling	MCL tear, LM tear, and a small tibial plateau fracture	Arthroscopic cleaning	4-week, 0–110° ROM, instability complaints persisted.

M, male; F, female; LM, lateral meniscus; BPTB, bone-patellar tendon-bone graft; ACL, anterior cruciate ligament; ROM, range of motion; MCL, medial collateral ligament.

**Table 2:** Published cases of pediatric anterior cruciate ligament femoral avulsion fractures.

Research	Publication	Year	Age	Sex	Approach	Surgical method
Robinson (12)	The Journal of Bone and Joint Surgery American Volume	1981	13	M	Medial parapatellar, lateral femoral	Pull-out sutures
Eady (13)	The Journal of Bone and Joint Surgery American Volume	1982	7	F	Medial parapatellar, lateral femoral	Pull-out sutures
Wasilewski (14)	The American Journal of Sports Medicine	1992	12	M	Medial parapatellar, lateral femoral	Pull-out sutures
Corso (15)	Arthroscopy: the Journal of Arthroscopic & Related Surgery	1996	3	M	Arthroscopy	Debridement, extension casting
Tohyama (16)	The American Journal of Sports Medicine	2002	11	M	Medial parapatellar, lateral femoral	Pull-out sutures
Kawate (17)	The Journal of Bone and Joint Surgery American Volume	2004	3	M	Arthroscopy, medial parapatellar	Pull-out wiring
Lakshmanan (18)	Knee Surgery, Sports Traumatology, Arthroscopy	2006	11	F	Arthroscopy, lateral femoral	Pull-out sutures
Rhee (19)	Injury Extra	2006	11	F	Physical therapy	/
Edwards (20)	Knee Surgery, Sports Traumatology, Arthroscopy	2007	11	M	Arthroscopy, medial parapatellar	Pull-out sutures
Bengtson (21)	Journal of Athletic Training	2011	10	M	Arthroscopy, medial parapatellar	Pull-out sutures
Wardle (22)	Annals of the Royal College of Surgeons of England	2012	11	F	Arthroscopy,	Pull-out sutures
Pai (23)	Case Reports in Medicine	2012	11	M	Arthroscopy, medial parapatellar	Pull-out sutures
Langenhan (24)	Strategies in Trauma and Limb Reconstruction	2013	14	F	Arthroscopy	K-wire fixation
Samuelsson (25)	BMJ Case Reports	2018	11	M	Physical therapy	/
Hasegawa (26)	JBJS Case Connector	2021	14	M	Arthroscopy	Modified Kessler sutures
Czer (27)	JBJS Case Connector	2022	10	M	Arthroscopy, lateral femoral	Pull-out sutures
Zheng (28)	Medicine	2022	11	F	Arthroscopy	Two no. 2Ethibond sutures

M, male; F, female.

## Case report

### Case characteristics

An 18-year-old healthy female experienced a traffic accident resulting in severe swelling and intense pain in her right knee. Initially, she sought treatment at a local hospital, where she underwent a computed tomography (CT) and magnetic resonance imaging (MRI) scans. Two days later, she was transferred to our hospital for further evaluation and treatment. The patient displayed an inability to bear weight. Physical examination revealed significant swelling and limited range of motion in the right knee. Positive Lachman and anterior drawer tests suggested an ACL injury, while the posterior drawer test was negative. Due to the severe knee pain and limited mobility, lateral stress tests, grind tests, and McMurray tests could not be performed to assess other potential injuries.

### Imaging

**Figure 1:**
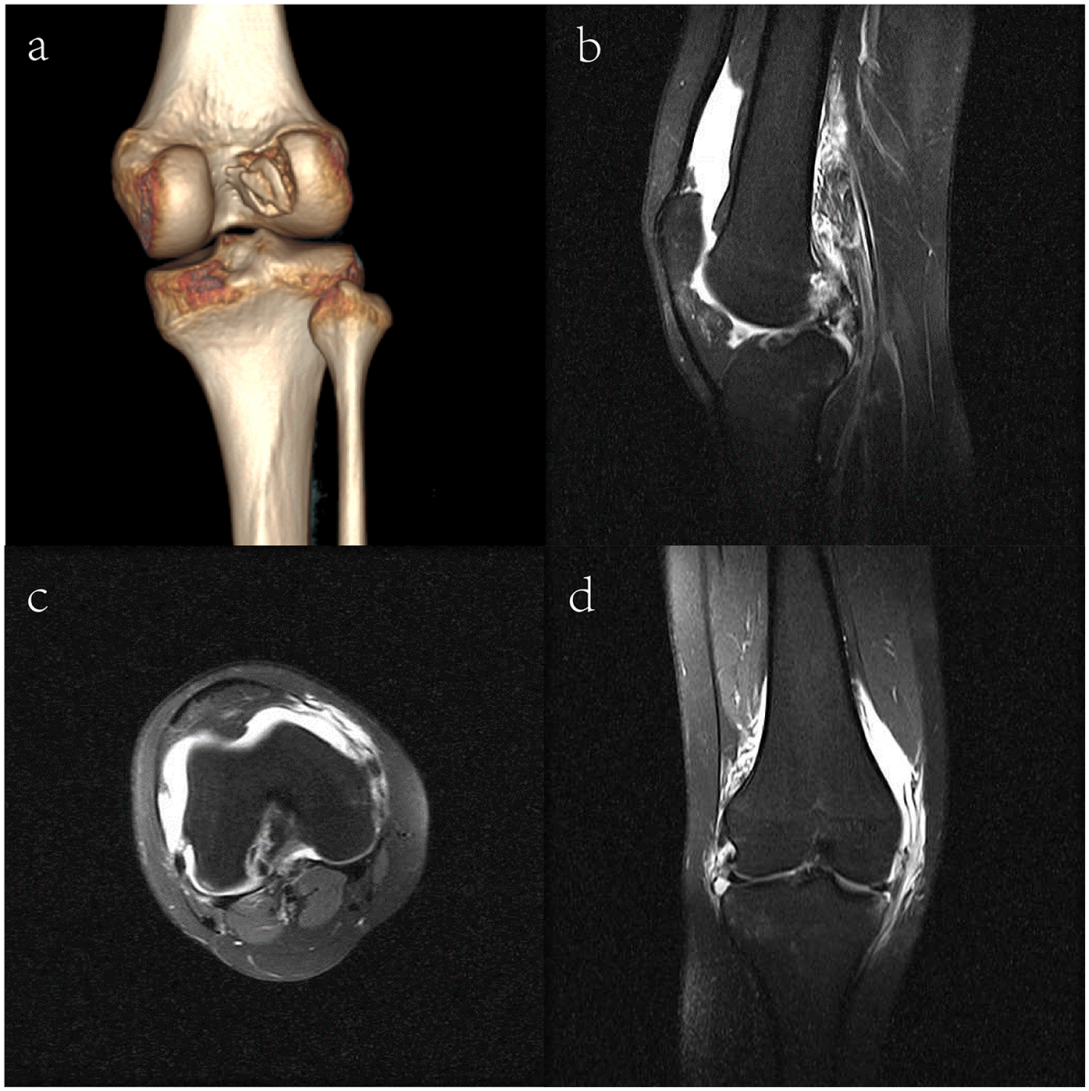
Imaging results of the patient. **(a)** Three-dimensional computed tomography scan; **(b)** Coronal view of the MRI; **(c)** Axial view of the MRI; **(d)** Sagittal view of the MRI.

The CT scan revealed discontinuity of the medial bone cortex at the lateral femoral condyle, with clear separation of the fracture fragments, consistent with an ACL femoral avulsion fracture (Figure 1a). MRI demonstrated significant joint effusion within the knee, confirming the avulsion fracture at the femoral attachment of the ACL and a rupture of the MCL, with no significant meniscal damage observed (Figures 1b-d). These imaging findings were essential for confirming the diagnosis and guiding treatment planning.

### Treatment strategy

**Figure 2:**
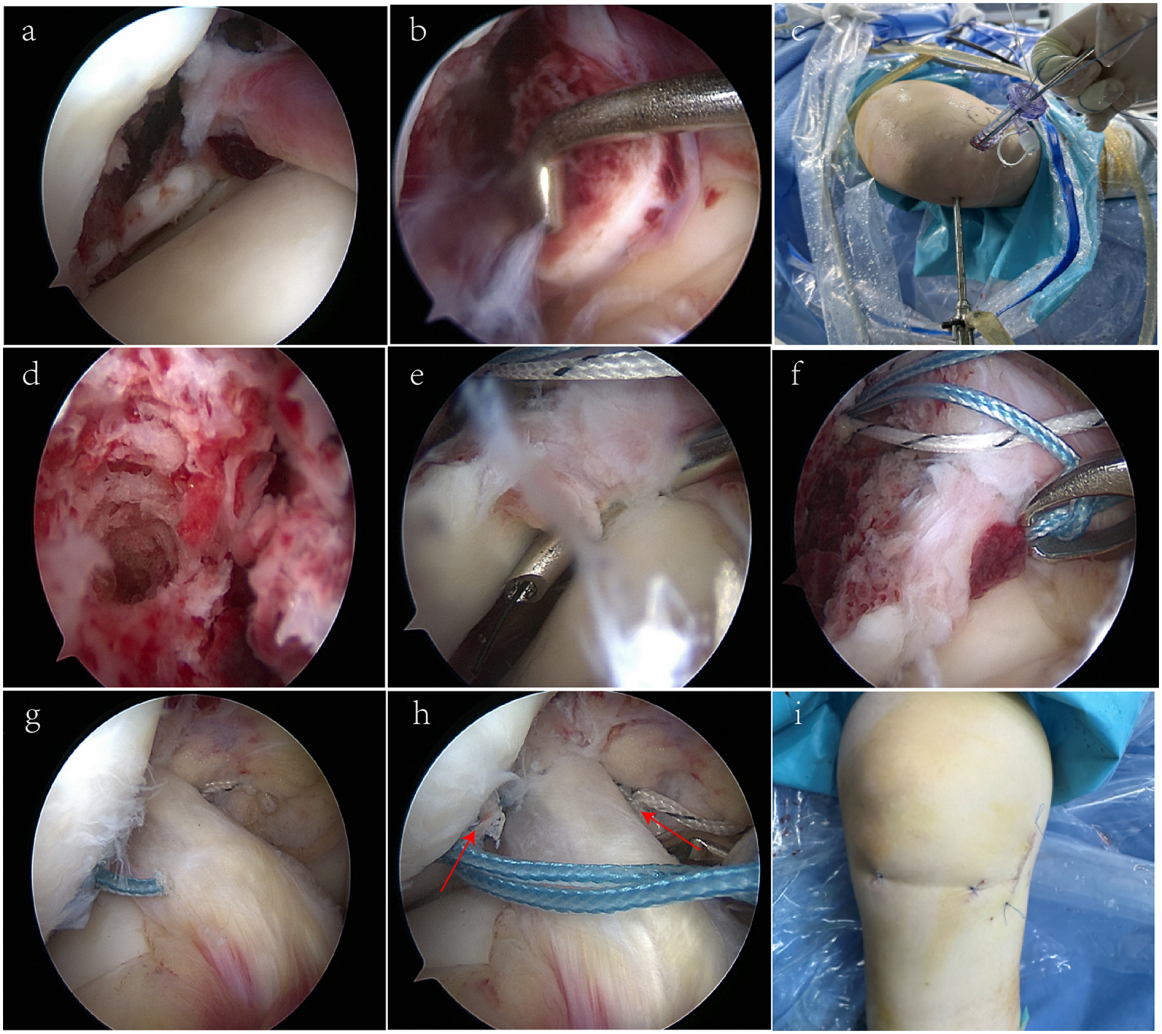
Arthroscopic fixation of ACL femoral avulsion fracture. **(a,b)** ACL with bone fragment; no cartilage damage. **(c)** 8 mm sheath insertion. **(d)** Pilot hole drilling. **(e,f)** Blue suture placement for PL bundle. **(g,h)** Final position of blue and white stitching. **(i)** Wound suturing.

After reviewing the patient's medical history, physical examination findings, and imaging results, and after thorough discussions with the patient and her family regarding treatment options and associated risks, the decision was made to proceed with arthroscopic surgery to repair the fracture. The surgery was performed four days after the injury. Under general anesthesia, a thorough examination of the knee joint revealed positive Lachman and anterior drawer tests (Supplementary Video 1). The arthroscopic examination showed a lax ACL with a significant bone fragment at the femoral attachment and no significant damage to the meniscus and articular cartilage (Figures 2a,b and Supplementary Video 2).

Fixation followed the principles of the anterior and posterior ACL bundles, specifically the posterolateral (PL) and anteromedial (AM) bundles (29). Initially, an 8 mm sheath was inserted through a medial incision in the knee (Figure 2c). Next, a pilot hole was drilled at the site of the femoral avulsion fracture of the ACL (Figure 2d). An absorbable composite bone anchor (Arthrex, Inc., Naples, FL, USA) was then placed into the pilot hole. Using a suture hook, the blue suture was threaded downwards to secure the PL bundle of the ACL on the femoral side, along with the bone segment on the inner lower side of the lateral femoral condyle (Figures 2e,f). Similarly, the white suture was used to secure the AM bundle of the ACL on the femoral side and the bone segment on the inner upper side of the lateral femoral condyle. The stability of the ACL was observed intraoperatively (Figures 2g,h and Supplementary Video 3).

A longitudinal incision of approximately 3 cm was made over the medial femoral condyle, passing through the skin and fascia to expose the attachment point of the MCL on the medial femoral condyle. Exploration revealed a complete rupture of the MCL at its femoral attachment, with signs of ligament contusion at the tear ends. The ligament was then sutured and repaired using a 1-0 synthetic absorbable suture. Finally, the wound was closed (Figure 2i). Postoperatively, a drawer test was conducted to assess joint stability (Supplementary Video 4).

### Postoperative course

Given the rarity of this type of injury, there are currently no standardized institutional protocols in place. Therefore, we decided to adopt our standard anterior ACL rehabilitation regimen. Postoperatively, the patient wore a hinged knee brace. Beginning on postoperative day one, quadriceps isometric exercises and straight leg raises were recommended. For the first four weeks, partial weight-bearing ambulation with full knee extension was recommended while using the brace. At the two-week mark, the patient undergoes a session of passive knee bending to 90 degrees under the guidance of a physiotherapist. Six weeks post-surgery, the patient begins formal rehabilitation training.

Clinically, after three months, the patient should be able to walk without the need for a brace. At the six-month post-operative follow-up, the patient demonstrated full knee range of motion (ROM) with no deformities or joint laxity during physical activities (Supplementary Video 5). Lachman and other stress tests were negative, indicating joint stability. A telephone follow-up conducted nine months after the surgery revealed that the patient was symptom-free and experienced no limitations in her physical activities. At the final 17-month follow-up, the knee remained stable and pain-free, with full ROM. Lachman, anterior drawer, and pivot shift tests were normal.

**Figure 3:**
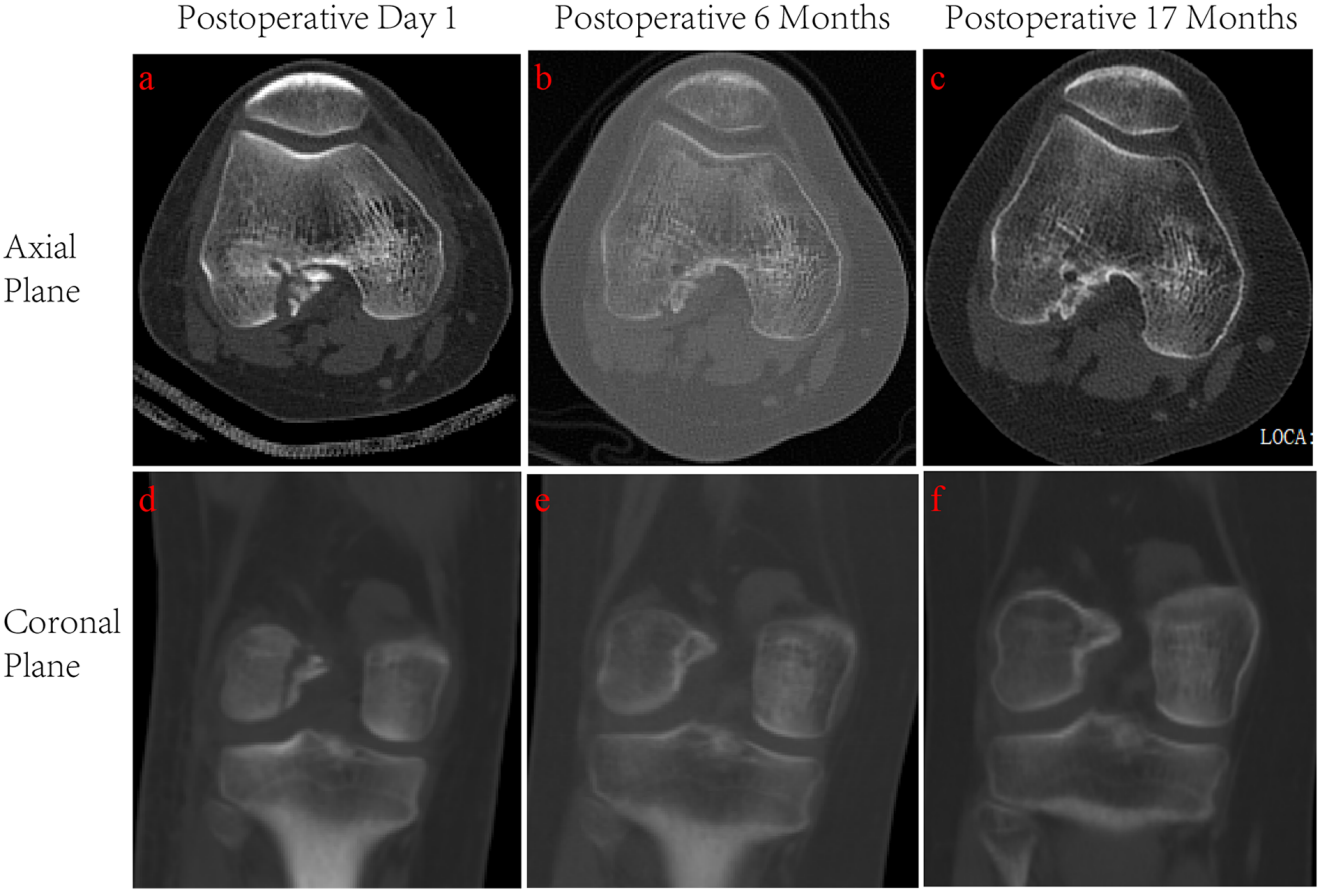
Postoperative CT imaging of the ACL femoral avulsion fracture at three follow-up intervals. **(a)** Postoperative Day 1, axial view. **(b)** 6-month follow-up, axial view. **(c)** 17-month follow-up, axial view. **(d)** Postoperative Day 1, coronal view. **(e)** 6-month follow-up, coronal view. **(f)** 17-month follow-up, coronal view.

Postoperative imaging evaluation was conducted using CT at three time points: Postoperative Day 1: Immediate CT imaging confirmed that the avulsed fragment was anatomically reduced, although a minimal residual gap was present at the fracture site (Figures 3a,d). 6-month follow-up: CT scans demonstrated complete osseous integration of the avulsed fragment, with trabecular bridging across the fracture line and no indications of nonunion (Figures 3b,e). 17-month follow-up: The final CT revealed full fracture consolidation, with uninterrupted continuity between the avulsed fragment and the femoral condyle, showing results similar to those observed at the 6-month follow-up (Figures 3c,f).

## Discussion

The ACL plays a crucial role in knee joint stability, and its injury can lead to anterior and rotational instability. Delayed or unsuccessful treatment may result in secondary traumatic arthritis, causing chronic knee pain and functional disabilities. While ACL tears are common in adults, femoral-side avulsion fractures are rare. These fractures are more prevalent in children due to their relatively stronger ligaments compared to bones and growth plates, whereas they are exceptionally rare in adults. To date, there have been only about 23 reported cases of femoral-side ACL avulsion fractures (Tables 1, 2). These injuries may result from both high-energy and low-energy traumas, typically involving acute flexion of the knee, internal rotation, and anterior tibial displacement relative to the femur. Movements that include valgus and external rotation of the tibia are also commonly implicated in these injuries.

In our case, the patient, who had just reached adulthood, suffered from an avulsion fracture at the ACL's femoral attachment, accompanied by a rupture at the MCL attachment point. The treatment goal for the ACL femoral avulsion fracture was anatomical reduction and stabilization of the bone fragments to facilitate the healing of the torn ACL, rather than ligament reconstruction. Previous reports have demonstrated favorable outcomes with surgical fixation. Currently, arthroscopic treatment of ACL femoral avulsion fractures represents a novel approach that minimizes surgical trauma, allows for rapid recovery, and achieves good functional results. The minimal scarring from this surgery is particularly advantageous aesthetically for young female patients. Given that this patient is a young female with a large bone fragment and the ligament was intact, fixation was achieved arthroscopically using a composite absorbable suture anchor. In the short term, we observed very good therapeutic outcomes.

In 2015, Nagaraj et al., Shah et al., and Prasathaporn et al. each reported initial case studies of adult avulsion fractures of the ACL at the femoral side (6–8). Nagaraj et al. described a 20-year-old male who sustained an ACL femoral avulsion fracture following a motorcycle fall (6). During arthroscopic surgery, displaced bone fragments were excised, and the ACL was reconstructed using a bone-patellar tendon-bone (BPTB) graft with bioabsorbable interference screws. At the three-month follow-up, the patient exhibited a negative Lachman test and a ROM from 0 to 135 degrees. Prasathaporn et al. reported a 25-year-old male who sustained an ACL femoral avulsion fracture after slipping, just six weeks after undergoing open fixation with plates and screws for a tibial plateau fracture (7). Due to complications on the tibial side, conventional ACL reconstruction was not feasible, so they opted to repair the ACL using suture anchor techniques. Six months post-operation, the patient's Lachman and anterior drawer tests were negative. Shah et al. reported a 47-year-old female who, while suffering an ACL femoral avulsion fracture, also had multiple fractures of the femoral and fibular shafts (8). They fixed the ACL using 4 mm cancellous screws and washers under arthroscopic guidance.

In 2016, Zabierek et al. reported on a 50-year-old male who sustained an ACL femoral insertion avulsion fracture during a knee pivoting incident while skiing (9). The patient underwent arthroscopic screw fixation and, four months post-operation, fully regained normal sports activity with excellent joint mobility. He performed the single-leg hop test without any limitations, and the strength of the quadriceps muscle on the injured side was comparable to the contralateral side. Both pivot shift and Lachman tests were normal.

Similarly, in 2018, Tharakulphan et al. reported a 32-year-old male who sustained an ACL femoral attachment avulsion fracture from a non-contact pivot injury during a soccer match (10). The treatment plan included using arthroscopic suture loop technique to fix the femoral attachment of the ACL avulsion fracture. Five months post-surgery, the patient reported no knee pain or swelling, and physical evaluations were negative for Lachman, anterior drawer, and pivot shift tests. Consequently, he was able to resume his pre-injury level of daily and sports activities.

In 2020, Brandsma et al. reported a 60-year-old female who sustained an avulsion fracture of the ACL at the femoral side, accompanied by a Grade 1 MCL tear and lateral meniscus damage (11). During arthroscopic surgery, only debris removal and meniscal reshaping were performed, with no ACL fixation. Despite regaining full function of her left knee four weeks post-surgery, the patient continued to experience instability. Consequently, conservative treatment for the ACL tear was resumed with the initiation of physical therapy to address this persistent instability.

In children and adolescents, the incidence of ACL femoral footprint avulsion fractures is higher than in adults, though still considered rare. Treatment strategies for this young patient group should focus on anatomical reduction and aim to preserve the native ACL as much as possible, due to the risk of valgus deformities following ACL reconstruction in young patients (30). For minors with incomplete skeletal development, extraphyseal repairs are essential to prevent further injury. Although some clinicians have employed percutaneous cross-pinning techniques, there have been no reports of malunion or asymmetric growth plate closure to date. Therefore, timely diagnosis and appropriate treatment are crucial to avoid complications such as deformity during healing (31).

Recently, in 2022, Zheng et al. reported an 11-year-old girl who sustained an avulsion fracture at the femoral insertion of the ACL (28). The case was managed with arthroscopic reduction and fixation using two No. 2 Ethibond sutures to secure the osteochondral fragments to the lateral femoral condyle. The sutures were brought out laterally and were secured proximally using a tie with an inside-out suturing technique. Twenty-four months post-operatively, the patient exhibited no pain, instability, or movement restriction. Both the Lachman test and pivot shift test were normal. This treatment method effectively minimized the risk of persistent ligament laxity and reduced complications associated with open surgery.

In 2021, Hasegawa et al. reported a 14-year-old boy who sustained a femoral avulsion fracture of both the anterior and posterior bundles of the ACL during a school sports activity (26). The patient underwent arthroscopic double-bundle pull-out repair surgery. A one-year follow-up indicated that the patient's knee joint was stable, without any deformities or laxity, and he was able to participate in various sports activities without restrictions.

In 2022, Czer et al. reported a 10-year-old boy who sustained an ACL femoral footprint avulsion fracture during a hiking trip (27). This case was treated using an arthroscopic dual-tunnel technique combined with a lateral femoral incision, which preserved the physeal plate. The 13-month follow-up demonstrated excellent radiological and clinical outcomes.

In our case, Postoperative CT imaging demonstrated a transient residual gap at the fracture interface on day 1, likely attributable to the physiological tension generated by the secured ACL fibers following anchor fixation. Despite this initial observation, progressive osseous integration was evident at the 6-month follow-up, with complete resolution of the residual gap and trabecular bridging confirming stable bone healing (Figure 3b). By 17 months, CT scans revealed full fracture union without displacement or degenerative changes, underscoring the long-term efficacy of the fixation (Figure 3c). The gradual degradation of the composite absorbable bone anchor facilitated load-sharing between sutures and healing bone, while controlled micromotion within the stable construct promoted secondary bone healing

In our cases, surgical intervention achieved successful anatomical reduction of the ACL femoral avulsion fracture, confirmed by immediate postoperative CT imaging, which revealed a transient residual gap at the fracture interface. This minimal gap likely resulted from physiological tension exerted by the secured ACL fibers following anchor fixation. Follow-up CT at 6 months demonstrated trabecular bridging and complete gap resolution, indicating stable healing with no signs of nonunion. By the 17-month follow-up, CT confirming sustained structural restoration. Clinically, the patient regained full knee range of motion within 8 weeks postoperatively and resumed unrestricted sports activities by 6 months, reporting no discomfort or functional limitations. Joint stability was maintained throughout follow-up, with negative Lachman, anterior drawer, and pivot shift tests. These outcomes underscore the efficacy of the arthroscopic anchor suture technique in achieving both anatomical restoration and functional recovery without requiring open arthrotomy.

## Conclusions

Although femoral avulsion fractures of the ACL are rare, physicians should be aware of them to ensure prompt and appropriate treatment considerations. In our case, the ACL femoral footprint avulsion fracture was precisely fixed with a composite absorbable bone equipped with suture anchors, achieving good clinical outcomes. The main advantages of this technique include the minimally invasive nature of the procedures, the promotion of anatomical reduction, the provision of stable fixation, and support for early resumption of patient activity. Future studies with larger sample sizes and higher-quality clinical trials are needed to determine the optimal surgical approach for such injuries.

## Data Availability

The original contributions presented in the study are included in the article/Supplementary Material, further inquiries can be directed to the corresponding authors.
